# The Inhibitory Effect of Extra Virgin Olive Oil and Its Active Compound Oleocanthal on Prostaglandin-Induced Uterine Hypercontraction and Pain—Ex Vivo and In Vivo Study

**DOI:** 10.3390/nu12103012

**Published:** 2020-09-30

**Authors:** Yi-Fen Chiang, Hui-Chih Hung, Hsin-Yuan Chen, Ko-Chieh Huang, Po-Han Lin, Jen-Yun Chang, Tsui-Chin Huang, Shih-Min Hsia

**Affiliations:** 1 School of Nutrition and Health Sciences, College of Nutrition, Taipei Medical University, Taipei 11031, Taiwan; yvonne840828@gmail.com (Y.-F.C.); ma07108002@tmu.edu.tw (H.-C.H.); hsin246@gmail.com (H.-Y.C.); a910241@gmail.com (K.-C.H.); phlin@tmu.edu.tw (P.-H.L.); jerris@tmu.edu.tw (J.-Y.C.); 2 Graduate Institute of Cancer Biology and Drug Discovery, College of Medical Science and Technology, Taipei Medical University, Taipei 11031, Taiwan; tsuichin@tmu.edu.tw; 3 Graduate Institute of Metabolism and Obesity Sciences, College of Nutrition, Taipei Medical University, Taipei 11031, Taiwan; 4 School of Food and Safety, Taipei Medical University, Taipei 11031, Taiwan; 5 Nutrition Research Center, Taipei Medical University Hospital, Taipei 11031, Taiwan

**Keywords:** primary dysmenorrhea, extra virgin olive oil, oleocanthal, mice writhing model

## Abstract

Primary dysmenorrhea is a common occurrence in adolescent women and is a type of chronic inflammation. Dysmenorrhea is due to an increase in oxidative stress, which increases cyclooxygenase-2 (COX-2) expression, increases the concentration of prostaglandin F2α (PGF2α), and increases the calcium concentration in uterine smooth muscle, causing excessive uterine contractions and pain. The polyphenolic compound oleocanthal (OC) in extra virgin olive oil (EVOO) has been shown to have an anti-inflammatory and antioxidant effect. This study aimed to investigate the inhibitory effect of extra virgin olive oil and its active ingredient oleocanthal (OC) on prostaglandin-induced uterine hyper-contraction, its antioxidant ability, and related mechanisms. We used force-displacement transducers to calculate uterine contraction in an ex vivo study. To analyze the analgesic effect, in an in vivo study, we used an acetic acid/oxytocin-induced mice writhing model and determined uterus contraction-related signaling protein expression. The active compound OC inhibited calcium/PGF2α-induced uterine hyper-contraction. In the acetic acid and oxytocin-induced mice writhing model, the intervention of the EVOO acetonitrile layer extraction inhibited pain by inhibiting oxidative stress and the phosphorylation of the protein kinase C (PKC)/extracellular signal-regulated kinases (ERK)/ myosin light chain (MLC) signaling pathway. These findings supported the idea that EVOO and its active ingredient, OC, can effectively decrease oxidative stress and PGF2α-induced uterine hyper-contraction, representing a further treatment for dysmenorrhea.

## 1. Introduction

Dysmenorrhea refers to severe pain in the lower abdomen and pelvis during menstruation [1]. This condition affects over 90% of women worldwide [2] and usually lasts for 24–72 h, and pain is the most critical symptom, which causes depression and a decline of quality of life [3]. The cause of dysmenorrhea may be an increase in intracellular calcium influx in uterine smooth muscle cells. Calcium influx induces extracellular signal-regulated kinase (ERK1/2) and activates phosphor–myosin light chain 20 (p-MLC20) [4,5]; furthermore, prostaglandin F2α (PGF2α) induces increased cyclooxygenase-2 (COX-2) [6]. The increase of COX-2 may be due to the increased oxidative stress, which causes pain [7]. The traditional treatment of primary dysmenorrhea is often in the form of non-steroidal anti-inflammatory drugs (NSAIDs), which are used to relieve pain or other accompanying symptoms [8,9,10]. NSAIDs, including ibuprofen, aspirin, and naproxen, are used to exert anti-inflammatory and analgesic antipyretic effects by inhibiting COX-2, but they have several side effects, including increased headaches, dizziness, nausea, and indigestion. Over long-term usage, patients may develop drug resistance [11].

As some serious side effects are present, researchers are eager to find a compound with a complementary effect to reduce the pain of this condition. Polyphenols, such as resveratrol and flavonoids in adlay hull extracts, can significantly reduce uterine contraction [12,13]. It has been shown that polyphenols or flavonoids may play an important role in reducing uterine contraction and reducing the pain of dysmenorrhea.

Polyphenol compounds may be found in different plants and fruits and have a potential anti-oxidant ability and anti-inflammatory effect [14]. The inhibition of oxidative stress is also related to the inhibition of uterine contraction [15,16]. Recently, oleocanthal—a natural compound from extra virgin olive oil—has been found to be the polyphenolic compound that causes throat irritation in extra virgin olive oil, and it can achieve anti-inflammatory effects by inhibiting COX-2. Moreover, it has ibuprofen-like activity [17], with a potential effect on pain-relief.

Extra virgin olive oil is one of the characteristic parts of the Mediterranean diet. It is produced in Spain, Italy, and Greece [18]. Extra virgin olive oil is rich in polyphenolic compounds, including oleocanthal, oleuropein, oleacein, and hydroxytyrosol, which have antioxidant abilities [19] and anti-inflammatory effects [20,21]. However, there is no research on oleocanthal’s effect on uterine contraction and its related molecular mechanism. In this study, we aim to demonstrate the effect of extra virgin olive oil’s polyphenolic compound, oleocanthal, on reducing uterine contraction.

## 2. Materials and Methods

### 2.1. Drugs and Solutions

The drugs and solutions used in this work were as follows: dimethyl sulfoxide (DMSO) (Sigma-Aldrich, St. Louis, MO, USA), oleocanthal (PhytoLab), acetonitrile, hexane, electrochemiluminescence (ECL) immunoassay (Thermo), oxytocin (Sigma-Aldrich, St. Louis, MO, USA, O3251) (Sigma-Aldrich, St. Louis, MO, USA, O6379), carbachol (Sigma-Aldrich, St. Louis, MO, USA, C4382), PGF2α (Cayman, Ann Arbor, MI, USA, 16010), acetylcholine (Sigma-Aldrich, St. Louis, MO, USA, A2661), Bay K 8644 (TOCRIS, Taiwan, 1544), potassium chloride (KCl) (SHOWA, Saitama, Japan, 1630-5150), calcium chloride dehydrate (CaCl_2_) (Wako, TX, USA, 038-00445), acetic acid (LAB-SCAN, Bangkok, Thailand, A8401E), β-Estradiol 3-benzoate (EB) (Sigma-Aldrich, St. Louis, MO, USA, E8515), ibuprofen (Sigma-Aldrich, St. Louis, MO, USA, I4883).

### 2.2. Experimental Animals

The experiment of uterine contraction used female Sprague Dawley rats (200–300 g), and the writhing test experiment used female Institute of Cancer Research (ICR) mice that were housed in a temperature-controlled room (22 ± 2 °C) with artificial illumination for 12 h, with food and water provided ad libitum. The study was approved by the Experimental Animal Care and Use Committee of Taipei Medical University (NO. LAC-2018-0210/NO. LAC-2018-0297). All animals received humane care in compliance with the Principles of Laboratory Animal Care and the Guide for the Care and Use of Laboratory Animals, published by the National Science Council, Taiwan.

### 2.3. Extra Virgin Olive Oil Extracts Preparation

The extra virgin olive oil (LAUDEMIO, L8057) was mixed with acetonitrile (ACN) and n-hexane (HEX) at a ratio of 1:2:3. After mixing, the solution was separated into ACN and HEX layers using a separator funnel. The ACN layer extracts after liquid separation were weighted and concentrated by a vacuum condenser. The ACN layer extract was concentrated to a small amount of liquid and transferred to a vial. It was then concentrated to a paste through continued rotation for 1 h for further experiments. after the layer forming the paste, the remaining liquid was aspirated. Finally, DMSO was used to dissolve the paste.

### 2.4. Determination of Extra Virgin Olive Oil (EVOO) Extracts by Reverse-Phase Ultra Performance Liquid Chromatography-Photodiode Array (UPLC–PDA) Analysis

A photodiode array (PDA) detector set to 223 nm was used for the identification of compound oleocanthal (OC), based on the retention time (Rt) and ultraviolet (UV) characteristics. A BEH Shieid RP18 column (Waters Acquity, 2.1 mm × 100 mm, 1.7 μm) was used for the analysis, and the flow rate was set to 0.5 mL/min. The mobile phase consisted of solvents A: acetonitrile (ACN) and B: water with 0.1% formic acid. The gradient method was used for the quantification of the compounds of interest for 0–5 min: 100% A; 0% B. The column temperature was kept at 40 °C throughout all experiments, and the injection volume was 10 μL.

### 2.5. Uterine Preparations and Measurement of Uterine Contraction

Rats were sacrificed by carbon dioxide and the uterus was removed and placed in an organ bath. The tissue water bath contained Krebs’ solution (113 mM NaCl, 4.8 mM KCl, 2.5 mM CaCl_2_, 18 mM NaHCO_3_, 1.2 mM KH_2_PO_4_, 1.2 mM MgSO_4_, 5.5 mM glucose, 30 mM mannitol, and pH 7.4). We removed the uterine fat and cut it into segments of equal length. The segments were placed in isolated organ baths containing Krebs’ solution at 37 °C with a 95% O_2_ and 5% CO_2_ supply. Each organ bath was equilibrated using 1 g of weight for at least 60 min. Uterine contractions were recorded with force-displacement transducers by using the LabScribe software [12].

### 2.6. Molecular Docking Site

Molecular docking was performed by a PyMOL plug-in—NRGsuite [22]—under the PyMOL version 1.8 environment. This included the detection of surface cavities in protein and was used as a target binding site for docking simulations. The structures of COX-2 bound with ibuprofen were downloaded from the protein data bank (PDB) database [23], and ibuprofen was separated from COX-2 in PyMOL for further docking analysis.

### 2.7. Acetic Acid-Induced Writhing Test

The test was carried out using a previously described technique [24]. Mice were treated with three different doses (28, 70, and 140 mg/kg) of extra virgin olive oil (EVOO) extracts and ibuprofen (120 mg/kg, P group) as a positive control. A total of 60 min after oral administration, writhing was induced by 0.6% acetic acid (10 mL/kg i.p., V group). Each mouse was placed in a transparent observation box, and the amount of writhing was recorded for 30 min after the acetic acid administration.

### 2.8. Oxytocin-Induced Writhing Test

This test was performed using the modified method described by a previous study [25]. During the experiment, the female mice were injected intraperitoneally with 1 mg/kg body weight estradiol benzoate and administration of EVOO extracts (28, 70, and 140 mg/kg) or ibuprofen (100 mg/kg, P group) every day, except for the control group, by an oral gavage. Mice were induced to writhe by an intraperitoneal injection with 67 IU/kg oxytocin solution in DMSO at day 7, and we recorded the number of writhing responses that occurred 30 min after oxytocin injection. Uterine samples were collected for protein expression analysis.

### 2.9. 2,2-Diphenyl-1-Picrylhydrazyl (DPPH) Assay

We used a commercial kit (Dojindo, Japan) and followed the instructions to evaluate the antioxidant ability. The scavenging activity was detected using 100 μL 2,2-Diphenyl-1-Picrylhydrazyl (DPPH) solution mixed with the sample in a 96-well microplate and incubated at room temperature for 30 min. The absorbance was measured at 517 nm using a VERSA Max microplate reader (Molecular Devices, San Jose, CA, USA) and using the following formula:

Scavenging activity (%) = [control − sample/control] × 100.

The IC_50_ DPPH values were obtained through extrapolation from regression analysis.

### 2.10. Lipid Peroxidation: Determination of Malondialdehyde (MDA)

The levels of malondialdehyde (MDA) in plasma followed the manufacturer’s instructions (TBARS Assay Kit Cayman, USA). Results were measured on a 532 nm plate using a VERSA Max microplate reader (Molecular Devices, San Jose, CA, USA).

### 2.11. Protein Preparation and Western Blot Analysis

To investigate whether OC affects the expression of PGF2α receptor (FP) protein in the uterus ex vivo, OC was added 10 min after the stimulation of the uterus with PGF2α, and the uterus was taken at 10, 30, and 60 min after the addition of OC for analysis.

Samples were lysed in radioimmunoprecipitation assay (RIPA) lysis buffer containing protease and phosphatase inhibitors (Roche, Mannheim, Baden-Württemberg, Germany). Protein was quantitated by the bicinchoninic acid assay (BCA) and then resolved using sodium dodecyl sulfate polyacrylamide gel electrophoresis (SDS-PAGE). After transfer to a polyvinylidene fluoride (PVDF) membrane, 5% bovine serum albumin (BSA) solution was used to block the empty space of the membrane. Subsequently, membranes were incubated with primary antibodies—PGF2α receptor (FP) (Cayman, 101802), COX-2 (Cayman, 160106), oxytocin receptor (OTR) (Santa Cruz, CA, USA, sc-8109), p-ERK (Cell Signaling, 9101), ERK (Cell Signaling, 9102), p-MLC20 (Cell Signaling, 3675), MLC20 (Santa Cruz, CA, USAsc-28329), Protein kinase C (PKC-δ) (BD Bio, 610397), α-actin (Santa Cruz, CA, USA, sc-32251)—at 4 °C overnight and then with a horseradish peroxidase (HRP)-conjugated secondary antibody (1:10,000) for 1 to 2 h. The visual signal was captured by an image analysis system (UVP BioChemi, Analytik Jena US, Upland, CA, USA). The band densities were determined as arbitrary absorption units using the Image-J software program version 1.52 *t* (NIH, Bethesda, MD, USA). The expression level of these target proteins was analyzed by three individual experiments.

### 2.12. Statistical Analysis

All data are presented as the mean ± standard error of the mean (SEM). The statistical significance of differences between the groups was analyzed by using Graphpad Prism (version 6.0). The statistically significant difference from the respective controls for each experiment was determined using a one-way analysis of variance (ANOVA) for all groups. A Student’s unpaired *t*-test was used for comparison between two groups. A value of *p* < 0.05 was considered to be statistically significant.

## 3. Results

### 3.1. Oleocanthal Inhibits Prostaglandin, Oxytocin, Acetylcholine, and Carbachol-Induced Uterine Hypercontraction Ex Vivo

**Figure 1:**
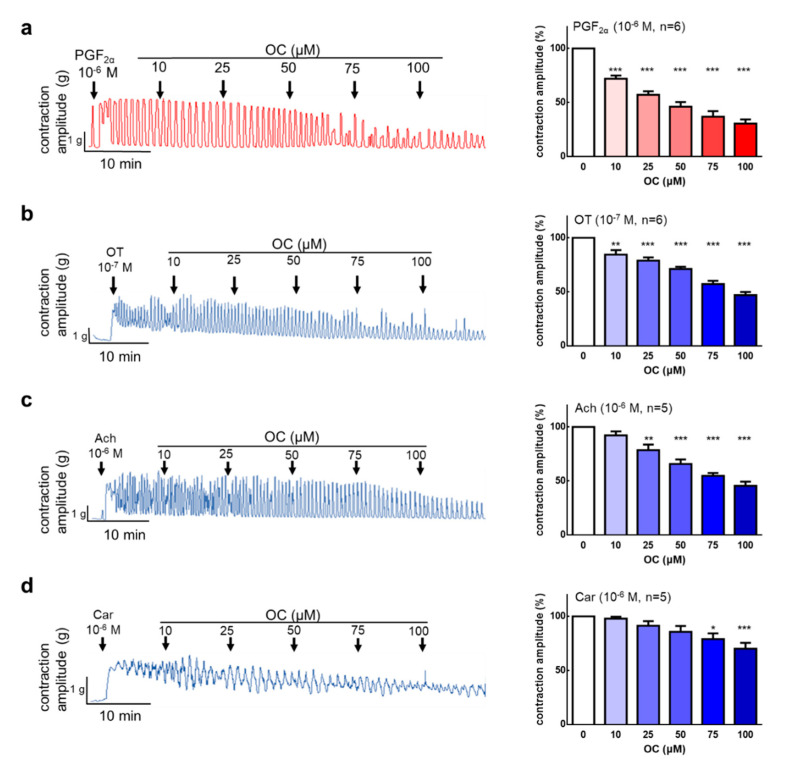
Effects of oleocanthal on prostaglandin F2α (PGF2α), oxytocin (OT), acetylcholine (Ach), and carbachol (Car)-induced uterine contraction in an ex vivo study. Oleocanthal (OC) (10–100 μM) was administered after (**a**) PGF2α-induced contractions, (**b**) OT-induced contractions, (**c**) Ach-induced contractions, and (**d**) Car-induced contractions. The effect of OC on the mean peak amplitude was calculated. Data are presented as the mean ± standard error of mean (SEM) (*n* = 3–6). * *p* < 0.05, ** *p* < 0.01, *** *p* < 0.001 versus 0 μM.

A pure compound of OC was used for ex vivo experiments. After the steady spontaneous contraction, PGF2α was used to induced hypercontraction. We also used PGF2α (10^−6^ M) to induce uterine contractions, and the uterus was treated with OC (10–100 μM). The results show that OC dose-dependently inhibited the PGF2α-induced contraction amplitude (Figure 1a, IC_50_ = 55.20 μM). The results demonstrate that OC has an effect on the inhibition of PGF2α-induced uterine hypercontraction. Oxytocin is a hormone that increases uterine and mammary gland contractions. OC (10–100 μM) was administered after oxytocin (10^−7^ M)-induced uterine contraction. The results show that OC can significantly inhibit oxytocin-induced uterine contractions (Figure 1b, IC_50_ = 91.35 μM). To investigate the effect of OC on the inhibition of acetylcholine or carbachol-induced uterine contraction, OC (10–100 μM) was added after acetylcholine or carbachol (10^−6^ M)-induced uterine contraction. OC can significantly inhibit acetylcholine (Figure 1c, IC_50_ = 85.63 μM) and carbachol (Figure 1d)-induced uterine contraction. Further analysis shows that the frequency had the identical effect in the uterine contract inhibition (Figure S1). To examine the OC’s cytotoxicity, it was treated with OC at the beginning and the organ bath was washed to exclude the OC’s effect, then it was treated with PGF2α. The results show that the organ can recover the contraction and that there is no cytotoxicity effect on the OC treatment. (Figure S2).

### 3.2. Effect of Oleocanthal on Ca^2+^-Dependent Contractions

**Figure 2:**
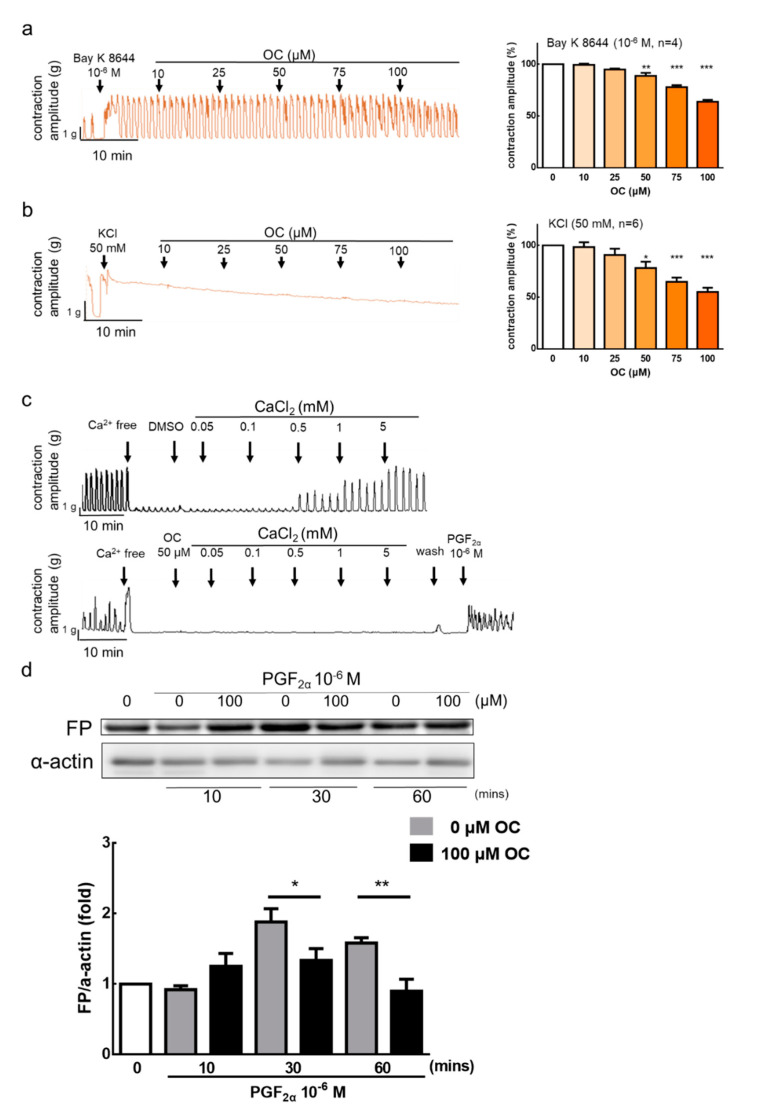
Inhibitory actions of oleocanthal on Ca^2+^-dependent contractile responses. Oleocanthal (OC) (10–100 μM) was administered after (**a**) Bay K 8644 and (**b**) potassium chloride (KCl). The effect of OC on the mean peak amplitude was calculated. Data are presented as the mean ± standard error of mean (SEM) (*n* = 3–6). * *p* < 0.05, ** *p* < 0.01, *** *p* < 0.001 versus 0 μM. (**c**) Ca^2+^-free medium containing the vehicle (0.2% dimethyl sulfoxide (DMSO)) or OC 50 μM for 60 min, and calcium (0.05–5 mM) was then cumulatively applied to trigger muscle contraction. We determined the cytotoxicity of oleocanthal by removing the oleocanthal and washing the tissue with Krebs’ solution, then reanalyzing the tissue in the presence of a stimulus (10^−6^ M prostaglandin F2α (PGF2α)). (**d**) PGF2α receptor protein expression was set at different time points in rat uterus by treatment with oleocanthal (100 μM). Data are presented as the mean ± standard error of mean (SEM) (*n* = 5). * *p* < 0.05, ** *p* < 0.01, versus vehicle group. FP: Prostaglandin F receptor.

Previous studies have reported that high K^+^ concentrations and Ca^2+^ activator can induce uterine contractions [26]. The results show that OC (50–100 μM) can inhibit Bay K 8644 (10^−6^ M), a Ca^2+^ channel activator, and KCl (50 mM)-induced uterine contraction (Figure 2a,b). In order to investigate the role of calcium, the following experiment used calcium to induce uterine contractions. When the solution without calcium was used, spontaneous uterine contractions were not present; after adding calcium (0.05–5 mM) into the solution, the spontaneous uterine contractions were restored (Figure 2c). However, when adding OC (50 μM) before adding calcium, the spontaneous uterine contractions were inhibited. This indicates that OC inhibits uterine contraction by blocking calcium channels. After removing OC and using PGF2α stimulant, the spontaneous contraction recovered by no more than 50% (Figure 2c). The results showed that, after treatment with OC, the intracellular calcium influx cannot effectively trigger uterine contractions. Therefore, we used a western blot to analyze the mechanism of OC in terms of the inhibition of uterine contraction. The results also show that OC inhibits the FP receptor to reduce uterine hypercontraction (Figure 2d). To examine the solvent effect, the solvent DMSO was treated after PGF2α oxytocin-induced contraction. Results found there are no effect on PGF2α, oxytocin, or calcium-induced uterine contraction inhibition (Figure S3). The inhibition effect of OC was not from the DMSO effect, and 0.04–0.4% DMSO shows no toxicity to the uterine.

### 3.3. Molecular Docking Site

**Figure 3:**
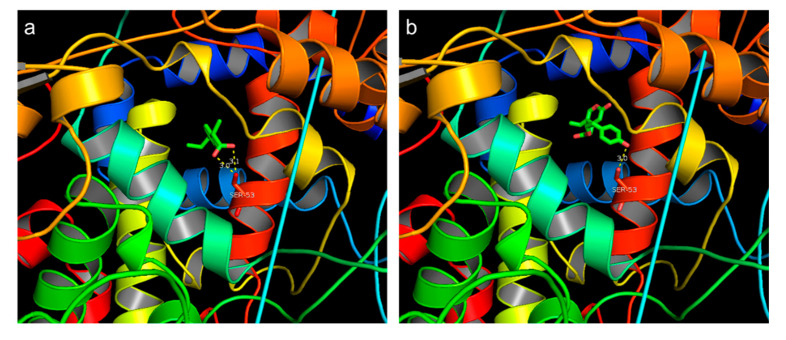
Molecular docking results of (**a**) ibuprofen and (**b**) oleocanthal compared to cyclooxygenase-2 (COX-2). The dashed line represents hydrogen bonds and distance (Å).

We proposed a hypothetical binding mode for oleocanthal and ibuprofen to the possible active site of human COX-2 (PDB ID: 4ph9), based on a computational docking program. Oleocanthal has been reported as a “natural ibuprofen”, which is a homolog of ibuprofen [17]. The docking results revealed that both oleocanthal and ibuprofen bind to Ser531 through hydrogen bonds and occupy the same as of COX-2 (Figure 3). This suggests that both compounds may have a similar effect to the COX-2 protein.

### 3.4. Extra Virgin Olive Oil Extraction Component and Inhibition Effect on Uterine Hypercontraction

#### 3.4.1. Extraction Rate of Extra Virgin Olive Oil

**Figure 4:**
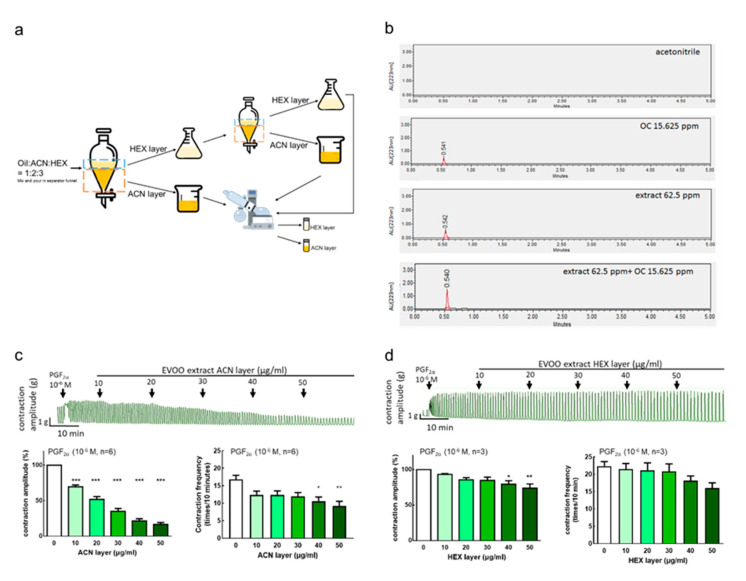
Effects of extra virgin olive oil (EVOO) extracts on prostaglandin F2α-induced uterine contraction in rats. The representative recordings, mean peak amplitude, and mean peak frequency of prostaglandin F2α (PGF2α)-induced contractions are presented. (**a**) The EVOO extraction process. (**b**) ultra-performance liquid chromatography (UPLC) of acetonitrile, oleocanthal at 15.625 ppm, and extra virgin olive oil ACN layer extracts at 62.5 ppm, as well as the mixture with oleocanthal at 15.625 ppm and extra virgin olive oil Acetonitrile (ACN) layer extracts at 62.5 ppm. (**c**) EVOO ACN layer extracts (10–50 μg/mL) and (**d**) EVOO Hexane (HEX) layer extracts (10–50 μg/mL). Data are presented as the mean ± standard error of mean (SEM) (*n* = 3–6). * *p* < 0.05, ** *p* < 0.01, *** *p* < 0.001 versus 0 μg/mL. OC: Oleocanthal.

To evaluate the effect of olive oil, we used an extraction of olive oil. The extracts were extracted with ACN and HEX, and the average extraction ratio of the ACN layer extracts was 0.18% in this study (Figure 4a).

#### 3.4.2. Extra Virgin Olive Oil Extraction and Oleocanthal Ultra Performance Liquid Chromatography (UPLC) Analysis Results

To investigate whether the ACN extracts contained the active ingredient, OC, we performed an analysis by UPLC. The data showed that the retention time of OC is 0.54 min, which is consistent with the ACN layer extracts. The higher the concentration, the larger the area under the curve, and thus we used a concentration range from 7.8125 to 62.5 ppm to establish a standard curve (Figure S4). The retention time of the EVOO ACN layer extracts was also 0.54 min. The area under the curve was embedded in the OC standard curve. When the EVOO ACN layer extracts of the extra virgin olive oil was 62.5 ppm, the OC contained about 13 ppm. When the EVOO ACN layer extracts was 125 ppm, the OC contained about 28 ppm, and the results show that OC accounted for about 20–22% of the EVOO ACN layer extracts. The OC and the ACN layer extracts, containing the same OC concentration, were combined and analyzed. The results showed that the retention time was 0.54 min, and the area under the curve was about twice the concentration of OC, further confirming that the ACN layer extracts contained OC (Figure 4b).

#### 3.4.3. Inhibition Effect of Extra Virgin Olive Oil Extracts on Prostaglandin-Induced Uterine Hypercontraction Ex Vivo

To examine the effect of olive oil extraction on uterine contraction, after inducing the contraction of the rat uterus by PGF2α (10^−6^ M), an EVOO ACN layer extract (10–50 μg/mL) or HEX layer extract (10–50 μg/mL) was added. The results showed that the addition of 10–50 μg/mL ACN layer extracts significantly inhibited the uterine contraction amplitude dose-dependently and significantly inhibited the uterine contraction frequency at 20–50 μg/mL (Figure 4c). On the other hand, 40–50 μg/mL HEX layer extracts significantly inhibited the uterine contraction amplitude (Figure 4d). This indicates that the active ingredient in EVOO, which can inhibit uterine contraction, is in the crude extracts of the ACN layer.

### 3.5. Inhibition Effect of Extra Virgin Olive Oil Extracts on Writhing Response Induced by Acetic Acid/Oxytocin in Female ICR Mice

**Figure 5:**
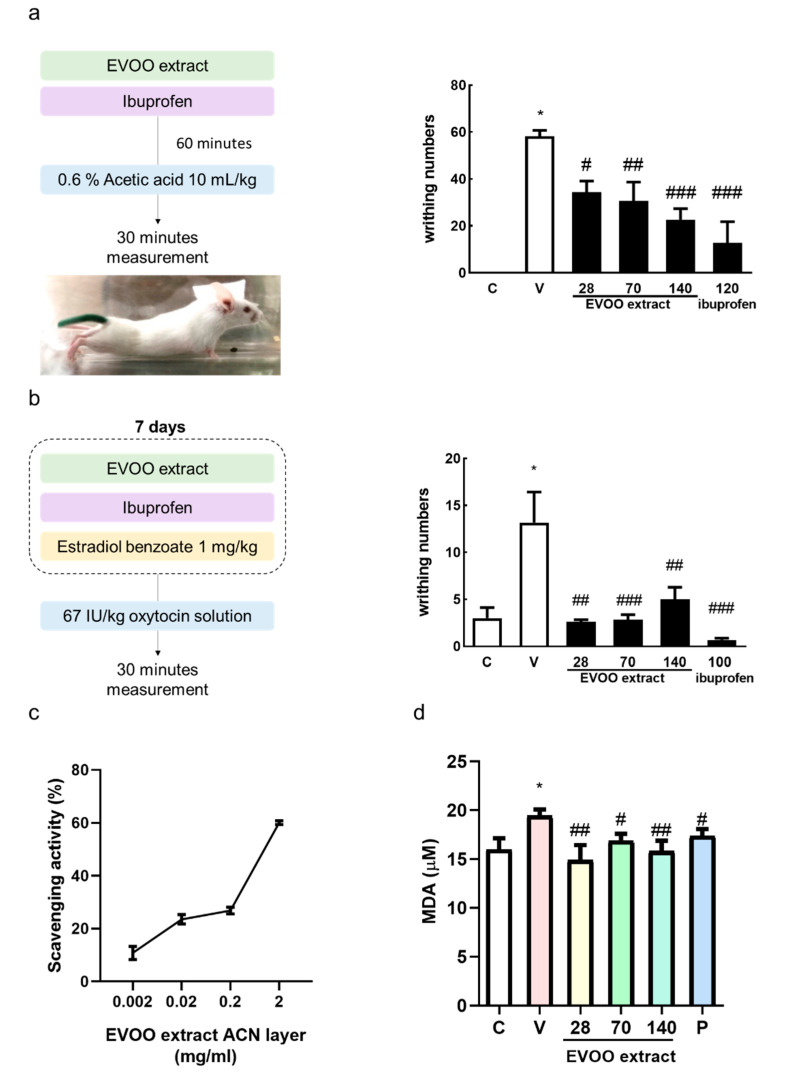
Effect of extra virgin olive oil (EVOO) extracts on acetic acid or oxytocin-induced writhing responses in mice. Mice were treated with EVOO extracts and ibuprofen (mg/kg, oral gavage), and we used (**a**) acetic acid injection, except for the control group, or (**b**) oxytocin injection. The amount of writhing was counted for 30 min after acetic acid administration. (**c**) 2,2-Diphenyl-1-Picrylhydrazyl (DPPH) scavenging activity and (**d**) malondialdehyde (MDA) concentration. Data are presented as the mean ± standard error of mean (SEM) (*n* = 5). * *p* < 0.05 versus control group, # *p* < 0.05, ## *p* < 0.01, ### *p* < 0.001 versus vehicle group. C, Control; V, Vehicle (67 IU/kg oxytocin). ACN: Acetonitrile.

**Figure 6:**
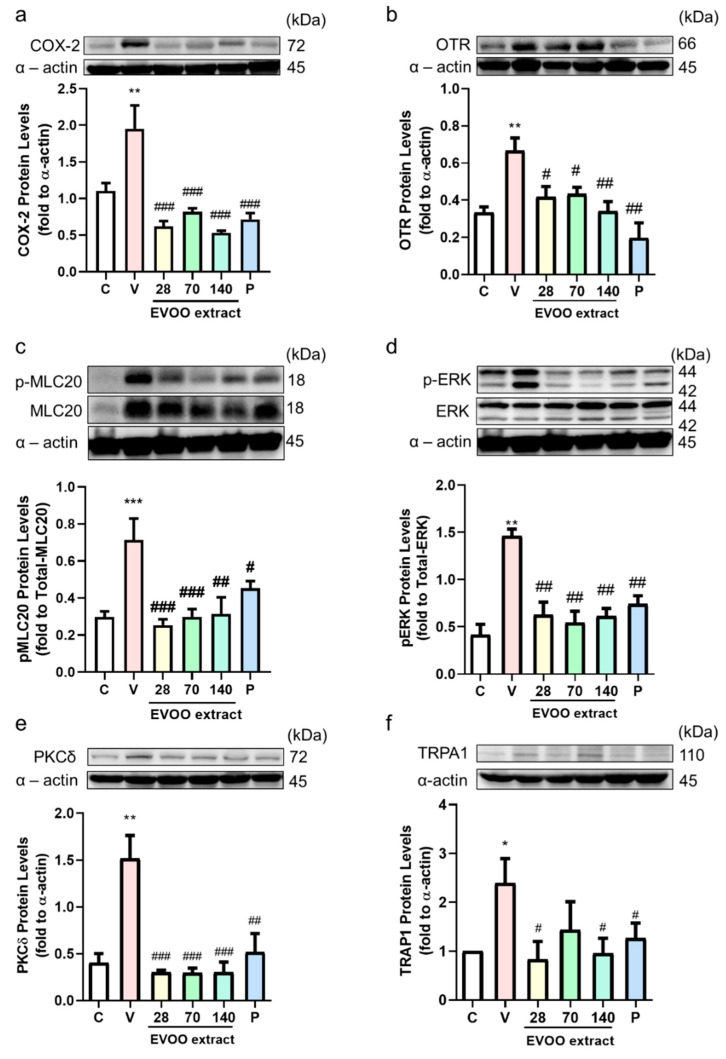
Extra virgin olive oil (EVOO) extracts modulated uterine contraction-related protein expression. Mice were treated with EVOO extracts (28, 70, 140 mg/kg oral gavage) or the same volume of vehicle or ibuprofen each week before oxytocin injection. After 30 min, we collected uterine samples for the Western blot test. (**a**) cyclooxygenase-2 (COX2), (**b**) oxytocin receptor (OTR), (**c**) phosphor–myosin light chain 20 (p-MLC20), (**d**) p-ERK, (**e**) PKCδ, and (**f**) Transient receptor potential ankyrin 1 (TRPA1) protein expression. C, control; V, vehicle (oxytocin-induced) P, ibuprofen 120 mg/kg. * *p* < 0.05; ** *p* < 0.01; *** *p* < 0.001 compared with C group. # *p* < 0.05; ## *p* < 0.01; ### *p* < 0.001 compared with V group. OTR: ERK: extracellular signal-regulated kinase, MLC20: myosin light chain 20, PKCδ: protein kinase C-δ.

The previous results of this study show that OC effectively inhibits the effects of uterine contractions. Pain is the most common symptom of dysmenorrhea, so we investigated the effect of extra virgin olive oil extracts on female ICR mice to inhibit the writhing reaction induced by acetic acid (Figure 5a) or oxytocin (Figure 5b). In both models, the amounts of writhing in the EVOO ACN layer extract groups were significantly lower than the control group, indicating that the EVOO ACN layer extracts had an analgesic effect. The EVOO ACN layer also showed an antioxidant ability by receiving a hydrogen ion from the ACN layer, turning the violet DPPH to yellow DPPH [27] (Figure 5c, IC_50_: 1.54 mg/mL). The plasma also showed that the treatment of OC can significantly reduce the MDA concentration (Figure 5d). Moreover, OC can significantly decrease oxytocin-induced COX-2 protein expression by decreasing OTR and transient receptor potential ankyrin 1 (TRPA1). Furthermore, it decreases OTR-modulated signaling transduction by decreasing the PKC-δ and ERK phosphorylation. Simultaneously, OC decreased the TRPA1-modulated calcium influx signal by decreasing the TRPA1, and the decreased calcium influx induced phosphorylation-MLC20 protein expression (Figure 6). 

## 4. Discussion

Dysmenorrhea has been reported to cause an increase in the production of prostaglandins, including PGF2α and of prostaglandin E2 (PGE_2_), which may cause the contraction of the myometrium. In addition, studies have reported higher levels of PGF2α in patients with primary dysmenorrhea [28]. An increase of COX-2 can increase the PGF2α in dysmenorrhea [29]. Meanwhile, during a contraction, the calcium influx will activate the phosphorylation of myosin light chain 20 (MLC-20) to control the relaxation or contraction of smooth muscle [12]. In an oxytocin-induced writhing animal model, the OTR can significantly increase COX-2 expression to increase the uterine contractions [30]. The increase of COX-2 may be induced by oxytocin-activated PKC and the phosphorylation ERK signaling pathway [31]. In uterine smooth muscle, oxytocin binds with the G protein-coupled receptor (GPCR) and produces calcium influx and diacylglycerol (DAG), which activates protein kinase C (PKC). PKC can activate several signaling pathways, such as extracellular signal-regulated kinase (ERK), to stimulate PGF2α synthesis [32].

In the search for a natural compound that has NSAID-like effects, some compounds have been discovered. In vivo and in vitro studies have shown that resveratrol can significantly reduce uterine contractions [12]. Moreover, in traditional Chinese medicine, adlay hull extracts also showed an effect on the inhibition of uterine contraction [13]. This shows that polyphenols may play an important role in reducing uterine contraction; furthermore, they can reduce the symptoms of dysmenorrhea. Extra virgin olive oil is produced in all countries in the Mediterranean region, including Spain, Italy, and Greece [18]. Previous studies have shown that high levels of extra virgin olive oil can inhibit platelet aggregation, lower cholesterol, and blood pressure, prevent cardiovascular disease and Alzheimer’s disease, and reduce arthritis, colon cancer, breast cancer, and prostate cancer risks [33,34,35]. The phenolic compound extract from extra virgin olive oil contains oleocanthal, which has been proven to have anti-inflammatory and anti-oxidant effects [20,21]. Oleocanthal is one of the compounds in EVOO causing irritation to the throat. Extra virgin olive oil produced in different regions has different oleocanthal concentrations, and extra virgin olive oil produced in Italy contains the highest concentration of oleocanthal (up to 191.8 ± 2.7 mg/kg). Laudemio’s extra virgin olive oil from Italy is abundant in oleocanthal content. Oleocanthal has an analgesic effect similar to ibuprofen, and long-term consumption may help prevent diseases. If the daily intake of 50 mg of extra virgin olive oil contains about 200 μg/mL oleocanthal (absorption rate is 60~90%), it can be taken up to 9 mg/day, which is equivalent to 10% of ibuprofen in an adult’s dose. However, the structure of oleocanthal is different from ibuprofen because of the analgesic effect of oleocanthal, which is also called “natural ibuprofen” [17]. The oral irritant, in a similar manner to ibuprofen, can nonspecifically modulate TRPA1 channel activation [36]. The expression of TRPA1 is strongly correlated with dysmenorrhea severity in endometriosis patients [37], and the activation of TRPA1 may trigger the calcium influx, while EVOO may decrease TRPA1 expression and calcium influx in Alzheimer’s disease [38].

This study is the first to demonstrate that extra virgin olive oil extracts and its active ingredient, oleocanthal, suppress PGF2α-induced uterine contractions in sprague dawley (SD) rats and can effectively inhibit pain in ICR mice. An EVOO ACN layer extract inhibited PGF2α induced uterine contractions in an ex vivo study; then, its active ingredient oleocanthal inhibited PGF2α, oxytocin, Ach, and carbachol-induced uterine contractions in rats. Oleocanthal also inhibited Bay K 8644, which is the calcium channel activator, and high K^+^ (KCl)-induced uterine contractions. In addition, oleocanthal can block PGF2α receptor to block Ca^2+^ influx through voltage-operated Ca^2+^ channels (VOCs). Finally, EVOO ACN layer extracts can effectively suppress pain in systemic pain or dysmenorrhea models in mice.

When PGF2α, oxytocin, carbachol, and acetylcholine are present, they will bind to an intracellular Gq-protein receptor and allow extracellular calcium to enter the cell via the calcium channel. When the cells are in a high potassium (>30 mM) environment, the VOC (L-type Ca^2+^ channel) will be turned on, allowing extracellular calcium to flow into the cells [39]. Our current study demonstrates that OC inhibits PGF2α, oxytocin, carbachol, or acetylcholine-induced uterine contractions, as well as Bay K 8644 or KCl-induced uterine contraction, possibly by reducing the calcium ion concentration and blocking calcium influx.

The main symptom of dysmenorrhea is lower abdominal pain. In order to simulate pain response, in the current study, we used acetic acid or oxytocin to induce a writhing reaction in ICR mice. The occurrence of writhing represents pain. The writhing reaction is characterized by abdominal wall contraction, pelvic rotation, and hind limb extension [40]. The acetic acid-induced writhing test is widely used to evaluate anti-inflammatory and analgesic activities. It increases the concentration of PGF2α and PGE_2_ in the peritoneal fluid and stimulates vasoconstriction, increasing the sensitivity of the peripheral nerves to prostaglandins [41]. In osteoarthritis, lipid peroxidation increases COX-2 and causes pain [7]. The antioxidant ability of OC reduces oxidative stress and lipid peroxidation, which reduces COX-2 protein expression and thus the pain. Oxytocin-induced writhing is the primary dysmenorrhea model; the uterus experiences ischemia, increasing the sensitivity of the distal nerve of the uterus to prostaglandins and then triggering the occurrence of a writhing reaction [31,42]. In this study, we confirmed that EVOO ACN layer extracts (28, 70, 140 mg/kg) effectively inhibit the writhing reaction induced by oxytocin compared with the control group. Furthermore, OC was shown to have an antioxidant ability, which reduced the COX-2 expression, decreased the oxytocin-induced related signaling pathway, and reduced the TRPA1 expression to decrease the calcium influx.

## 5. Conclusions

**Figure 7:**
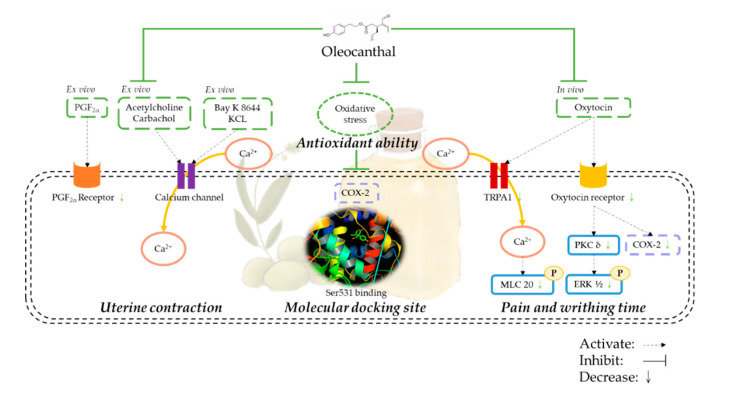
Schematic representation of the inhibitory effect of Oleocanthal (OC) on uterine hyper-contraction by the inhibition of calcium influx and the contraction signaling pathway and the use of a similar binding site to ibuprofen. PGF2α: prostaglandin F2α, KCL: potassium chloride, COX-2: cyclooxygenase-2, TRPA1: transient receptor potential ankyrin 1, MLC20: myosin light chain-20, PKCδ: protein kinase c-δ, ERK1/2: extracellular signal-regulated kinase 1/2.

According to the results, extra virgin olive oil ACN layer extracts and oleocanthal bind the same site of COX-2 as ibuprofen, resulting in an antioxidant ability, blocking calcium and reducing the TRPA1 channel and oxytocin receptors to inhibit uterine contractions by reducing the calcium influx and p-ERK and p-MLC20 signaling pathways to achieve an analgesic effect. Therefore, extra virgin olive oil and its active ingredient oleocanthal have the potential to improve primary dysmenorrhea (Figure 7).

## Supplementary Materials

The following are available online at https://www.mdpi.com/2072-6643/12/10/3012/s1. Figure S1: Effect of OC prostaglandin F2α (PGF2α), oxytocin (OT), acetylcholine (Ach), and carbachol (Car)-induced uterine contraction in an ex vivo study. Figure S2: Examine the cytotoxicity effect of oleocanthal. Figure S3: Effect of solvent DMSO on uterine contraction. Figure S4: Representative UPLC chromatography.
