# Supplementary material for: The Inhibitory Effect of Extra Virgin Olive Oil and Its Active Compound Oleocanthal on Prostaglandin-Induced Uterine Hypercontraction and Pain—Ex Vivo and In Vivo Study
Source: Nutrients. 2020 Sep 30;12(10):3012. doi: 10.3390/nu12103012 (PMC7599558; doi:10.3390/nu12103012)
Supplement: Supplementary file 1 [file nutrients-12-03012-s001.pdf]

The Inhibitory Effect of Extra Virgin Olive Oil and Its Active Compound Oleocanthal on Prostaglandin-Induced Uterine Hypercontraction and Pain—Ex Vivo and In Vivo Study

Yi-Fen Chiang [1], Hui-Chih Hung [1,†], Hsin-Yuan Chen [1], Ko-Chieh Huang [1], Po-Han Lin [1], Jen-Yun Chang [1], Tsui-Chin Huang [2] and Shih-Min Hsia [1,3,4,5,*]

[1] School of Nutrition and Health Sciences, College of Nutrition, Taipei Medical University, Taipei 11031, Taiwan; yvonne840828@gmail.com (Y.-F.C.); ma07108002@tmu.edu.tw (H.-C.H.); hsin246@gmail.com (H.-Y.C.); a910241@gmail.com (K.-C.H.); phlin@tmu.edu.tw (P.-H.L.); jerris@tmu.edu.tw (J.-Y.C.)

[2] Graduate Institute of Cancer Biology and Drug Discovery, College of Medical Science and Technology, Taipei Medical University, Taipei 11031, Taiwan; tsuichin@tmu.edu.tw

[3] Graduate Institute of Metabolism and Obesity Sciences, College of Nutrition, Taipei Medical University, Taipei 11031, Taiwan

[4] School of Food and Safety, Taipei Medical University, Taipei 11031, Taiwan

[5] Nutrition Research Center, Taipei Medical University Hospital, Taipei 11031, Taiwan

* Correspondence: bryanhsia@tmu.edu.tw; Tel.: +886-2-2736-1661 (ext. 6558)

† Equal contribution as first author.

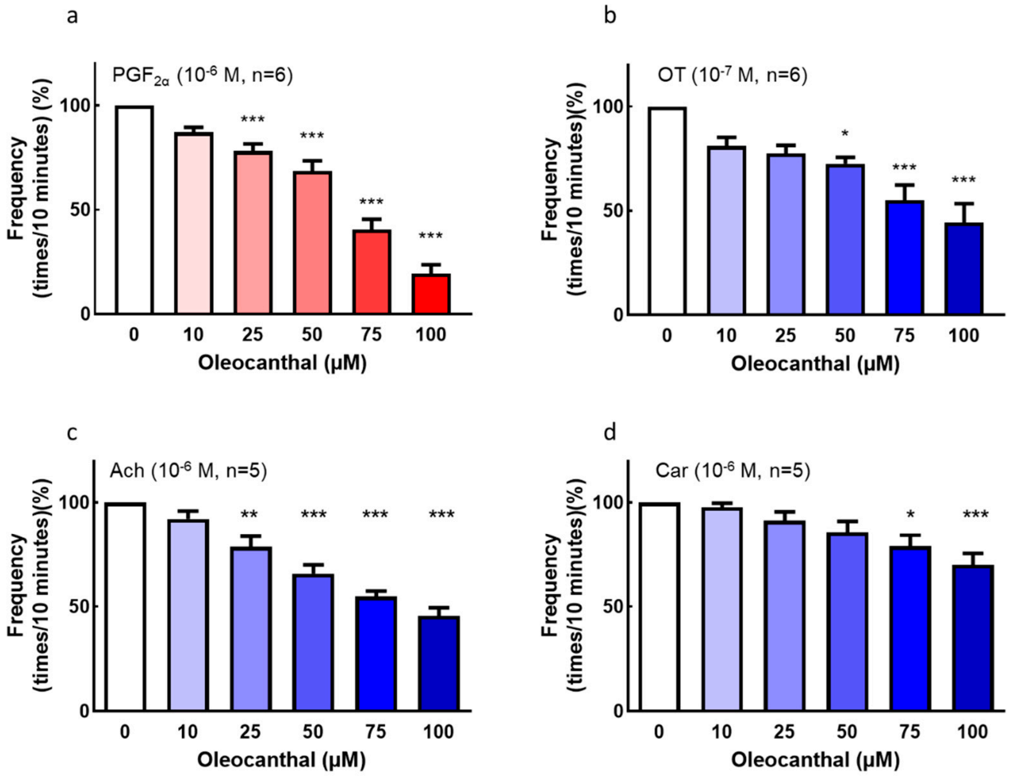

Figure S1. Effect of OC prostaglandin F2α (PGF2α), oxytocin (OT), acetylcholine (Ach) and carbachol (Car)-induced uterine contraction in an *ex vivo* study.

(a) $PGF_{2\alpha}$ (b) OT (c) Ach (d) Car induced contraction. The effect of OC on the frequency was calculated. Data are presented as the mean ± SEM (n=3-6). *$p < 0.05$, **$p < 0.01$, ***$p < 0.001$ versus 0 μM.

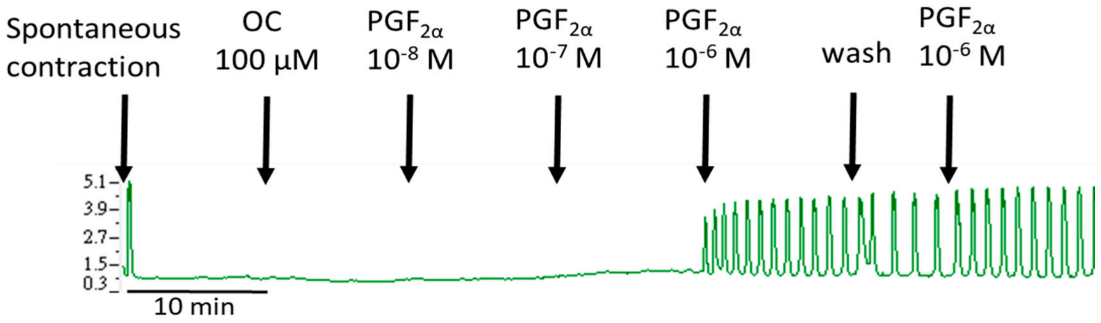

Figure S2. Examine the cytotoxicity effect of oleocanthal.

Uterine was placed to perform spontaneous contraction and treated with oleocanthal. After wash uterine can successfully induced again after oleocanthal treatment. Showing no cytotoxicity effect in oleocanthal treatment.

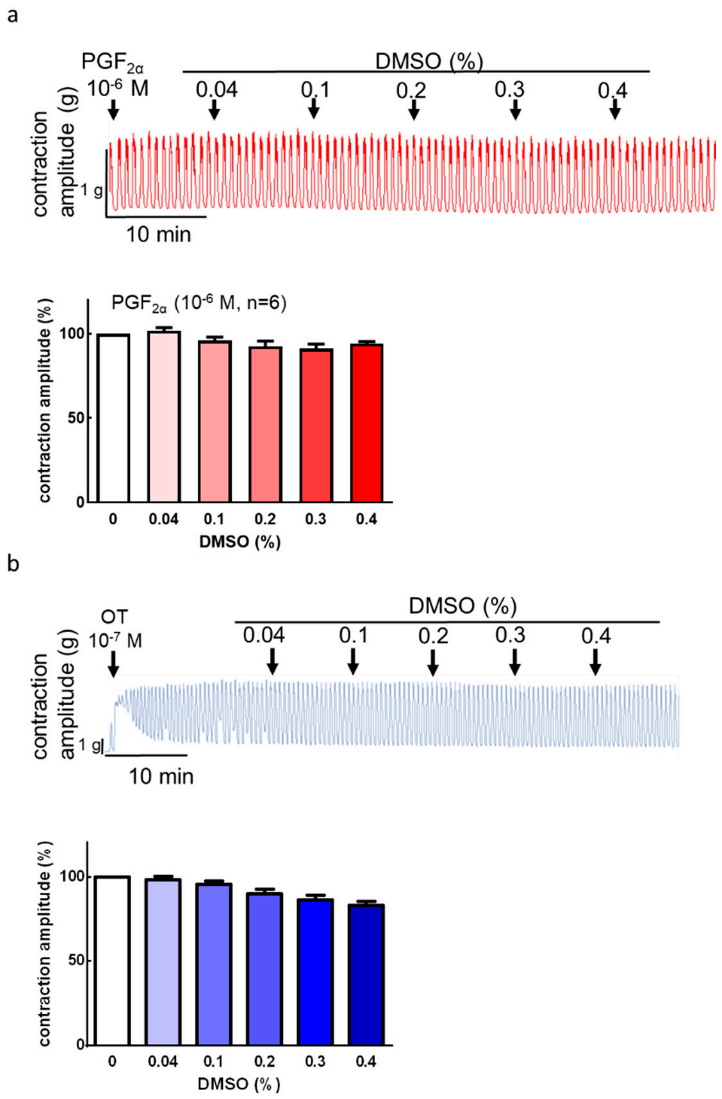

Figure S3. Effect of solvent DMSO on uterine contraction. (a) $PGF_{2\alpha}$-induced contractions. (b) Oxytocin induced contraction.

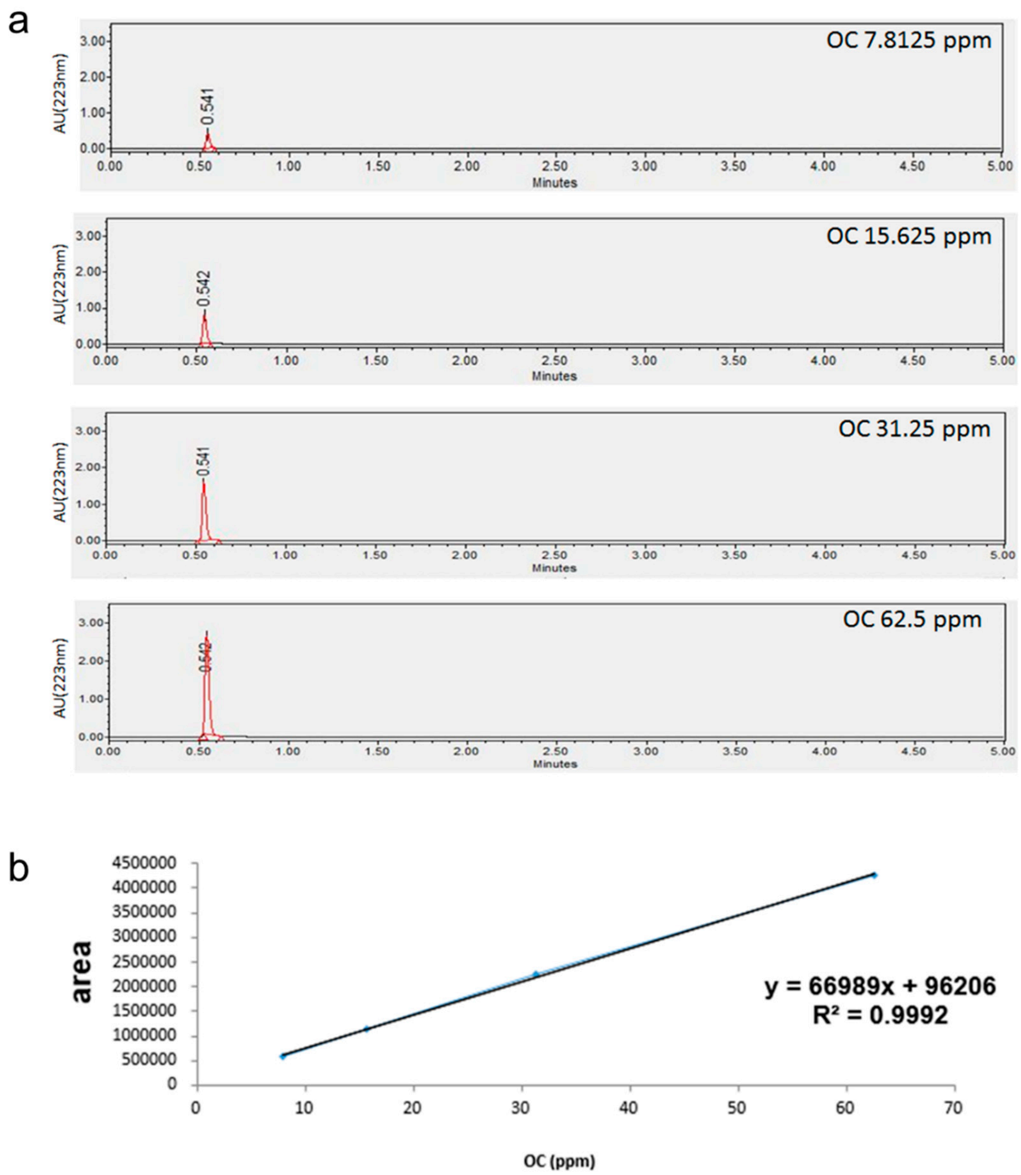

Figure S4. Representative UPLC chromatography of (a) oleocanthal from 7.8125 to 62.5 ppm. (b) UPLC chromatography of oleocanthal standard curve
